# Conceptual DFT-Based Computational Peptidology of Marine Natural Compounds: Discodermins A–H

**DOI:** 10.3390/molecules25184158

**Published:** 2020-09-11

**Authors:** Norma Flores-Holguín, Juan Frau, Daniel Glossman-Mitnik

**Affiliations:** 1 Laboratorio Virtual NANOCOSMOS, Departamento de Medio Ambiente y Energía, Centro de Investigación en Materiales Avanzados, Chihuahua, Chih 31136, Mexico; norma.flores@cimav.edu.mx; 2 Departament de Química, Universitat de les Illes Balears, E-07122 Palma de Malllorca, Spain; juan.frau@uib.es

**Keywords:** Discodermins A–H, Chemical Reactivity Theory, Conceptual DFT, Global and Local Reactivity Descriptors, pKa, Bioavailability, Bioactivity scores, ADME

## Abstract

A methodology based on the concepts that arise from Density Functional Theory named Conceptual Density Functional Theory (CDFT) was chosen for the calculation of some global and local reactivity descriptors of the Discodermins A–H family of marine peptides through the consideration of the KID (Koopmans in DFT) technique that was successfully used in previous studies of this kind of molecular systems. The determination of active sites of the studied molecules for different kinds of reactivities was achieved by resorting to some CDFT-based descriptors like the Fukui functions as well as the Parr functions derived from Molecular Electron Density Theory (MEDT). A few properties identified with their ability to behave as a drug and the bioactivity of the peptides considered in this examination were acquired by depending on a homology model by studying the correlation with the known bioactivity of related molecules in their interaction with various biological receptors. With the further object of analyzing their bioactivity, some parameters of usefulness for future QSAR studies, their predicted biological targets, and the ADME (Absorption, Distribution, Metabolism, and Excretion) parameters related to the Discodermins A–H pharmacokinetics are also reported.

## 1. Introduction

Several natural resources in the sea may generate molecules which could be a lead toward the design of new medical drugs. It is for this reason that many recent studies have been undertaken towards new products where the knowledge of marine natural products is used as an important source of information [1]. Among the systems that can be obtained from natural products with marine origin are peptides, which are molecules with a size ranging between that of proteins and amino acids [2]. Peptides are sources of nitrogen and amino acids which are related to numerous potential physiological functions. Bioactive peptides can be protein fragments that acquire functionality when liberated from the parent protein. The first activity assigned to a peptide was neurotoxicity; however, at present, they are associated with other functions, such as cardiotonic, antiviral and antitumor, cardiotoxic and antimicrobial activity. These functions, in addition to their excellent binding properties, low off-target toxicity, and high stability, make peptides promising molecules for the development of new therapeutics. Approximately 60% of described natural products belong to the peptide family. Peptides are present in many marine species, and the extensive research that has been conducted on them has shown that they most often found in sponges, as occurred with polyketides, peptides can be also produced by commensal or symbiotic bacteria or fungi.

Due to the great potential that these peptides offer as an aid in the treatment of many diseases, they are commonly referred to as therapeutic peptides. As a consequence, there is a lot of current research in this field [2]. By considering these therapeutic peptides from the viewpoint of a medicinal angle, a detailed knowledge of their bioactivity as well as their chemical reactivity properties at the molecular level is of great interest. As it is well known, the chemical reactivity properties and bioactivity of these peptides are closely related from a molecular viewpoint [3,4]. For this reason, we are currently doing exhaustive research in this field by studying different families of marine peptides (mainly cyclodepsipeptides) trying to find the relationships that could help in the development of new medical drugs for fighting several diseases.

The process of drug design is driven by a combination of advanced experimental and computational methods. The use of Computational Chemistry methodologies has a very important role in the practice of modern medicinal chemistry, offering a great potential for the improvement of the different phases of drug research, with special emphasis on time and cost savings. Since the structure of molecules defines the chemical, physical and biological properties of matter, this information is crucial for understanding, explaining and predicting chemical reactions and biochemical processes, developing new drugs and providing important insights into the nature of the interactions between drug targets and ligands, which allows predictive models that are suitable for lead discovery and optimization to be constructed.

For this reason, it is of the utmost importance to study the chemical reactivity of the natural products (in this case of marine origin), because it is likely to help with the development of some medicines with the aid of tools displayed through Computational and Theoretical Chemistry as well as Molecular Modeling. In a special way, Conceptual Density Functional Theory (DFT) [5,6,7] is one of the most powerful tools within those that are available for the study, understanding and comprehension of the chemical reactivity of molecular systems. Conceptual DFT, sometimes known as Chemical Reactivity Theory, is able to help in the prediction of the relationships between the bioactivity and the chemical reactivity properties by considering a series of global and local descriptors that arise from the fundamentals of the method [8,9,10].

**Figure 1:**
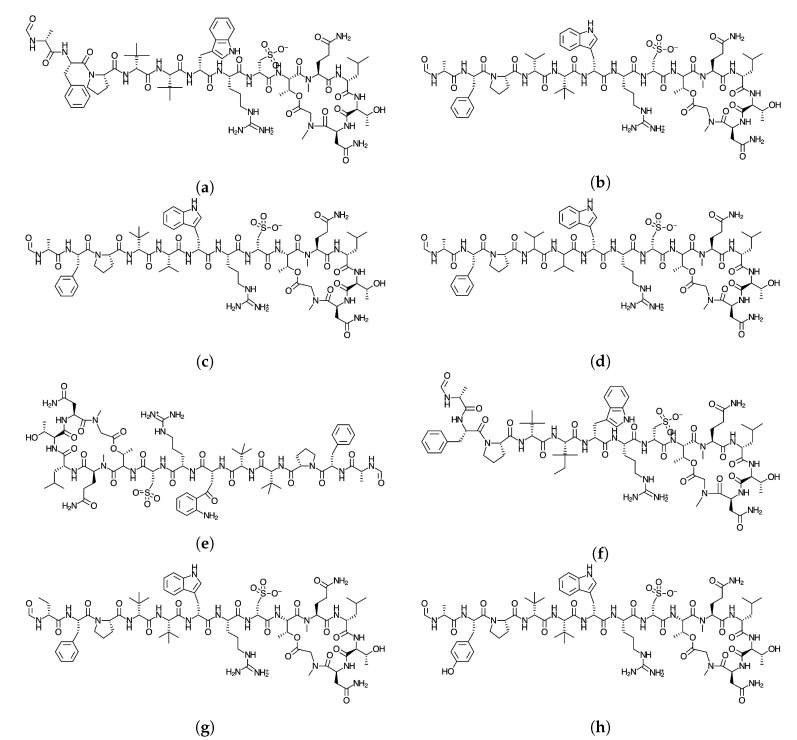
Graphical sketches of the molecular structure of (**a**) Discodermin A, (**b**) Discodermin B, (**c**) Discodermin C, (**d**) Discodermin D, (**e**) Discodermin E, (**f**) Discodermin F, (**g**) Discodermin G, and (**h**) Discodermin H.

Since the knowledge of the chemical reactivity is essential for the development of new pharmaceutical drugs, we are now investigating a new family of peptides obtained from marine origin with the hope that this could be a new source for therapeutic peptides [2]. The objective of this study Is to report the global and local chemical reactivity descriptors of the Discodermins A–H family of marine peptides whose graphical sketches are shown in Figure 1 by making use of the Conceptual DFT methodology. It also involves the determination of the potential reaction sites for the cases of nucleophilic and electrophilic attacks. By considering a methodology that we have developed and validated before, the prediction of the pKa values for each peptide has been attained [11]. The study has been complemented by considering the report of some additional properties that could be useful in QSAR involving several methodologies addressed in the literature [12,13], as well as a detailed study of bioactivity radars that can give an idea of the drug-like behavior of the studied peptides, the predicted biochemical targets on the basis of an homology methodology and the values associated with pharmacokinetics. By following this approach that we have coined as Conceptual DFT-based Computational Peptidology, as a branch of Computational Chemistry dedicated to the study of peptides, the current study acts as a follow-up to previously published results on some families of therapeutic peptides of marine origin [14,15,16,17,18]. it must be also mentioned that besides the Conceptual DFT descriptors that constitute an important ingredient of the proposed Computational Peptidology methodology, the chemical reactivity of a given system could also been successfully approached by resorting to the local approach of DFT through local softness theory [19].

## 2. Theoretical Background and Computational Details

Kohn–Sham (KS) methodology includes the estimation of the molecular energy and density of a given system, as well as the orbital energies, explicitly connected with the frontier orbitals including the Highest Occupied Molecular Orbital (HOMO) and Lowest Unoccupied Molecular Orbital (LUMO) [20,21,22,23]. This methodology is convenient when thinking of quantitative qualities related to Conceptual DFT descriptors. At present, the use of range-separated (RS) exchange-correlation density functionals in Kohn–Sham DFT is of extraordinary concern [24,25,26,27]. It is essential to the development of these density functionals to think about the partitioning of the exchange and the ${}_{12}^{-1}$ operator into long-and short-ranged parts partnering with a range-separation ω parameter that controls the rate at which long-range behavior is obtained. The estimation of ω can either be fixed or “tuned” by use of a molecule-by-molecule procedure by adhering to some tuning principles. The ideal tuning methodology depends on having the KS HOMO energy related to vertical ionization potential (IP) which is an estimation of the energy difference, E(N-1)-E(N). For the instance of the Generalized KS theory appropriate to an N-electron molecular system, we should have -IP(N) = $\epsilon_{H}$(N), which can be considered to be the DFT counterpart of the well-known Koopmans’ theorem. In reality, this is valid only for the exact density functional. For the situation where for pragmatic reasons, we have to consider an approximated density functional, there will be possibly some critical distinction between -IP(N) and $\epsilon_{H}$(N), Thus, ideal tuning involves setting up ω, system-specific range-separation parameter, by a nonempirical approach and having a RSE useful density functional [28,29,30,31,32,33,34,35]. Indeed, even with the absence of an equal methodology that can be used for the correlation of the electron affinity (EA) combined with the energy of the LUMO, it can be drawn that $\epsilon_{H}$((N + 1) = −EA(N) is conceivable. This makes the acquisition of the optimized ω value easier, provided that the differences between $\epsilon_{L}$(N) and $\epsilon_{H}$(N + 1) are small. Through this, the forecast of the Conceptual DFT descriptors expectation is upgraded for a given optimized density functional. The concurrent prescription is dubbed as the “KID procedure” (for Koopmans in DFT), this being in reference to the relationship it has with Koopmans’ theorem that has been previously quoted [14,15,16,17,18].

Following the methodology considered in our previous studies [14,15,16,17,18], we have done the computational determinations by using the Gaussian 09 series of programs [36] for the implementation of the density functional needed for the development of this work. The Def2SVP basis set [37,38] was chosen for the geometry optimizations and in the verification that the optimized structures corresponded to the minimal ones through the calculation of the associated frequencies. As a larger basis set is usually needed for the calculation and analysis of the electronic properties, the Def2TZVP basis set was chosen [37,38] as a constituent of our standard methodology. According to our previous studies on this kind of molecular systems, water was selected as the solvent through the Solvation Model Density (SMD) parameterization of the Integral Equation Formalism-Polarized Continuum Model (IEF-PCM) [39] for all the DFT calculations. For the determination of the molecular structures and their associated electronic properties of the studied peptides, the MN12SX density functional was chosen because it is already well known that it is capable of giving very good results for several structural and thermodynamic properties [40]. The resulting model chemistry, MN12SX/Def2TZVP/H20, was then considered model chemistry due to the excellent behavior that our previous research has demonstrated owing to the fact that the MN12SX behaves as a Koopmans-complaining density functional which is very useful for obtaining accurate HOMO and LUMO orbital energies, thus avoiding the determination of the energies of the cationic and anionic systems for which convergence is usually hard to obtain for the somewhat large molecules as peptides are [14,15,16,17,18].

## 3. Results and Discussion

**Figure 2:**
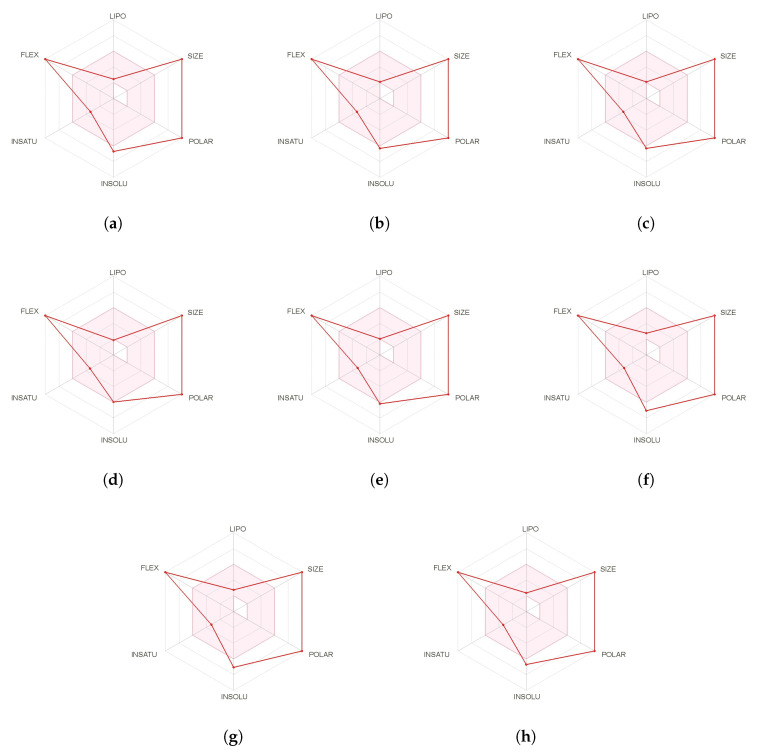
Bioavailability radars of the (**a**) Discodermin A, (**b**) Discodermin B, (**c**) Discodermin C, (**d**) Discodermin D, (**e**) Discodermin E, (**f**) Discodermin F, (**g**) Discodermin G and (**h**) Discodermin H molecules.

The starting molecular structures of the Discodermins A–H peptides to be studied were obtained from ChemSpider (http://www.chemspider.com/), which is a website that acts as an online free resource of chemical information related to physical and biological properties, interactive spectra and literature references. In order to get a glimpse of the potential therapeutic properties of the considered peptides, Simplified Molecular Input Line Entry Specification (SMILES) notations for the molecular systems under consideration were fed into the online Molinspiration software from Molinspiration Cheminformatics (Slovensky Grob, Slovak Republic) allowing for the estimation of several molecular properties that are known to be related to druggability and that could be useful in QSAR studies [41,42] that are commonplace in the process of drug design and development. The results of this determination are presented in Table 1:

A further and complementary step can be performed by resorting to SwissADME [43] which is an online free tool that allows the evaluation of drug-likeness through a graphical representation of the properties of interest called Bioavailability Radar which is obtained for every peptide by with the aid of its SMILES representation and where the pink area exhibits the zone with the optimal range for a particular property, as shown in Figure 2:

Another information that can be obtained from the precedent study is that related to the pharmacokinetic properties of the potential therapeutic peptides, i.e., how the living organism will interact with the drugs since they are delivered to the body up to when they or their metabolites are finally excreted. This information is collectively known as ADME properties, and this important information in the process of drug discovery is presented in Table 2 for the Discodermins A–H marine peptides.

**Figure 3:**
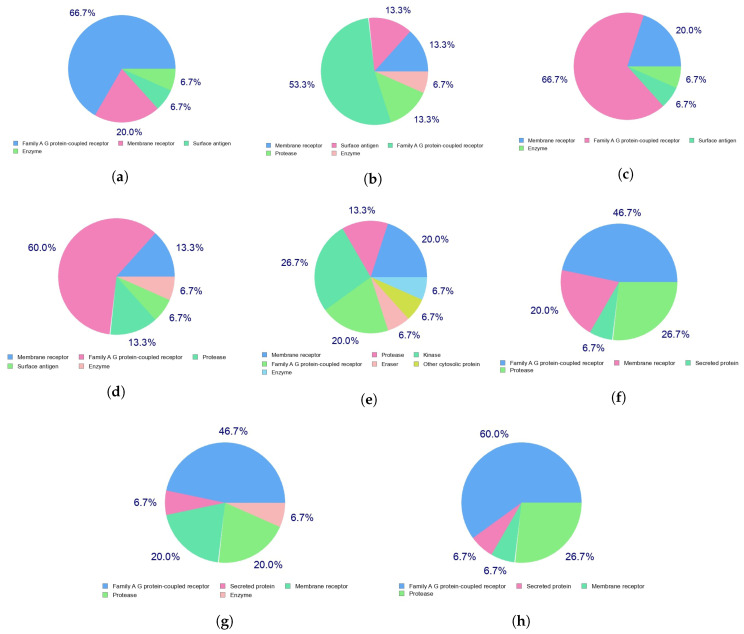
Predicted biological targets of the (**a**) Discodermin A, (**b**) Discodermin B, (**c**) Discodermin C, (**d**) Discodermin D, (**e**) Discodermin E, (**f**) Discodermin F, (**g**) Discodermin G and (**h**) Discodermin H molecules.

Indeed, the efficacy of a given therapeutic drug will be highly dependent on the way it interacts with a given receptor in the cells, which in turn with the diseases that the medicines will be designed to fight. This can be predicted beforehand by resorting to a homology procedure using databases of known molecules of similar and related structures to the ones under study. This is an important tool in the drug discovery process and has been used in this work to analyze the Discodermins A–H family of peptides by resorting to the free online SwissTargetPrediction software [44] with the results for the predicted biological targets presented in Figure 3.

All the presented results for the potential therapeutic activity of the Discodermins A–H family of peptides are an indication that the chemical reactivity of these molecules is an area worth to be explored. As we did in the past [14,15,16,17,18]. A methodology called Conceptual DFT-based Computational Peptidology developed in our research group will be considered now for the calculation and analysis of the chemical reactivity properties of these interesting molecules. The starting point consists of a search for the most stable conformers of each peptide on the basis of the molecular structures taken from the ChemSpider webpage by resorting to the MarvinView 17.15 program (ChemAxon, Budapest, Hungary) relying on the overall MMFF94 force field [45,46,47,48,49].

The most stable conformer for each peptide obtained through the described procedure were then subjected to a geometry optimization in the gas phase by considering the Density Functional Tight-Binding Approximation (DFTBA) model that is accessible in Gaussian 09 [36]. As it was mentioned in the Theoretical Background and Computational Details section, the resultant structures were subsequently reoptimized by considering the MN12SX/Def2SVP/H2O model chemistry that have proven to be adequate for this purpose [14,15,16,17,18]. Upon verification that every optimized structure corresponded to a minimum in the energy potential curve by employing the frequency-calculation analysis technique, the electronic properties were calculated by resorting to a similar model chemistry but considering the Def2TZVP basis set instead of the Def2SVP one because it has been demonstrated [14,15,16,17,18] that this is a better choice for the prediction and analysis of the HOMO and LUMO orbitals and of the chemical reactivity properties derived from them.

**Figure 4:**
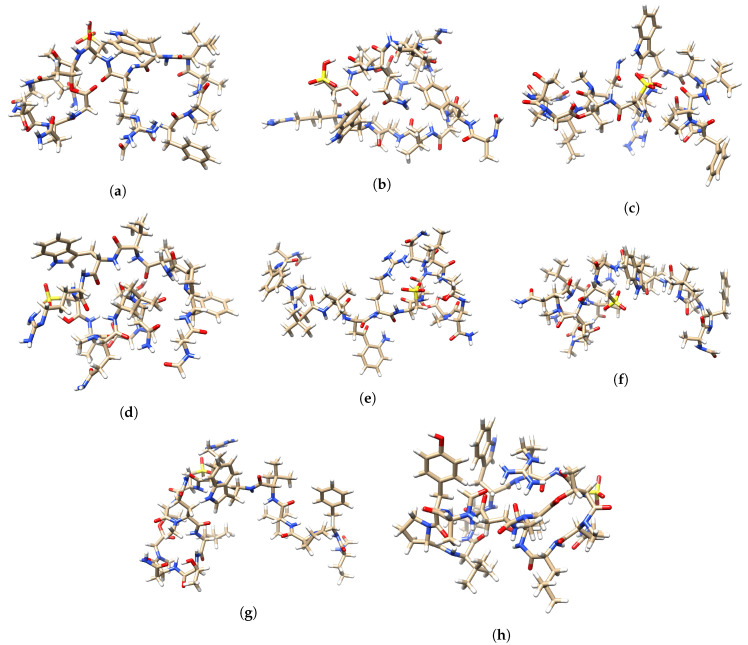
Optimized molecular structures of (**a**) Discodermin A, (**b**) Discodermin B, (**c**) Discodermin C, (**d**) Discodermin D, (**e**) Discodermin E, (**f**) Discodermin F, (**g**) Discodermin G and (**h**) Discodermin H.

A graphical display of the tridimensional optimized molecular structures of the Discodermins A–H peptides obtained with the aid of the UCSF Chimera Visualization System [50] are presented in Figure 4.

It is usually assumed that the goodness of a given density functional can be estimated by comparing the results that it gives with the experimental values that are trying to be reproduced or with the results that can be obtained through post Hartree–Fock calculations like MP2, MP4 or CCSD. However, this is not always possible due to the lack of experimental results for the molecular systems that are being studied or the large size of the molecules that keep some accurate methodologies to be computationally practical. For this reason, we have developed a protocol named KID (Koopmans in DFT) [14,15,16,17,18], which is an attempt to validate a given density functional in terms of its internal coherence. On doing it previously, several descriptors associated with the results that the HOMO and LUMO calculations obtained are related with results obtained using the vertical I and A following the ΔSCF procedure, where SCF refers to the Self-Consistent Field technique. It has been shown that there is a connection between the key descriptors and the simplest conformity to the theorem of Koopmans or the Ionization Energy theorem, which is its equivalent within the Generalized Kohn–Sham (GKS) version of DFT, by connecting $\epsilon_{H}$ to −I, $\epsilon_{L}$ to −A, and their actions by defining the HOMO—LUMO gap $J_{I}=\left|\epsilon_{H}+E_{gs}\left(N-1\right)-E_{gs}\left(N\right)\right|$, $J_{A}=\left|\epsilon_{L}+E_{gs}\left(N\right)-E_{gs}\left(N+1\right)\right|$, and $J_{HL}=\sqrt{{J_{I}}^{2}+{J_{A}}^{2}}$. It should be noticed that the $J_{A}$ descriptor consists of an approximation which is only valid if the HOMO of the radical anion (the SOMO) resembles the LUMO of the neutral system. For this reason, another descriptor ΔSL has been designed by our research group [14,15,16,17,18], to help in the verification of the accuracy of the approximation. Although the Koopmans’-complaining behavior of the MN12SX density functional has been proved previously for the case of peptides [14,15,16,17,18], we think that it is worth performing a further validation for the case of the molecules considered in the present study. This determination has been achieved by making use of the in-house developed CDFT software tool and the results of this analysis are shown in Table 3.

As can be seen from the results in Table 3, the values for the KID descriptors are all very close to zero which is an indication that the chosen MN12SX density functional behaves as a Koopmans-complaining one and that for that reason the MN12SX/Def2TZVP/H2O is a model chemistry that has been further demonstrated very adequate for the purpose of this research.

Taking into account the KID methodology considered in the previous research being integrated into the finite difference approximation [14,15,16,17,18], the following definitions can be used for the global descriptors that help in the understanding of the chemical reactivity of the molecular systems [5,6,7,51,52]:
Electronegativity$\chi =-\frac{1}{2}\left(I+A\right)\approx \frac{1}{2}\left(\epsilon_{L}+\epsilon_{H}\right)$Global Hardness$\eta =\left(I-A\right)\approx \left(\epsilon_{L}-\epsilon_{H}\right)$Electrophilicityω = $\frac{\mu^{2}}{2\eta }=\frac{{\left(I+A\right)}^{2}}{4(I-A)}\approx \frac{{\left(\epsilon_{L}+\epsilon_{H}\right)}^{2}}{4(\epsilon_{L}-\epsilon_{H})}$Electrodonating Power$\omega^{-}$ = $\frac{{\left(3I+A\right)}^{2}}{16(I-A)}\approx \frac{{\left(3\epsilon_{H}+\epsilon_{L}\right)}^{2}}{16\eta }$Electroaccepting Power$\omega^{+}$ = $\frac{{\left(I+3A\right)}^{2}}{16(I-A)}\approx \frac{{\left(\epsilon_{H}+3\epsilon_{L}\right)}^{2}}{16\eta }$Net Electrophilicity$\Delta \omega^{\pm }=\omega^{+}-\left(-\omega^{-}\right)=\omega^{+}+\omega^{-}$
being $\epsilon_{H}$ and $\epsilon_{L}$ the HOMO and LUMO energies associated with each of the peptides considered in this work. It is worth mentioning that for the global indices the chemical power is directly related with the electronic density as well as the corresponding Hohenberg-Kohn functional [53].

As a complement of these global reactivity descriptors that arise from Conceptual DFT [5,6,7,51,52], Domingo and his collaborators [54,55,56,57,58] have proposed a Nucleophilicity index N through the consideration of the HOMO energy obtained through the KS scheme with an arbitrary shift of the origin taking the molecule of tetracyanoethylene (TCE) as a reference.

By making use of the mentioned CDFT software tool applied to the results of the calculation of the electronic properties of the Discodermins A–H peptides, the values of the defined global reactivity descriptors (including the Nucleophilicity N) could be obtained and they are displayed in Table 4:

The Global Hardness η can be seen as a measure of the resistance of the electronic density to be deformed and thus as an indication low reactivity of a given molecular system. From the results of Table 4, it can be concluded that Discodermin E will be the more reactive peptide in this family, while Discodermin G and Discodermin H will be the less reactive ones, being the chemical reactivity of the other peptides approximately the same. An analog behavior is observed for the Electrophilicity ω descriptor which encompasses the balance between the tendency of an electrophile to acquire an extra number of electrons and the resistance of a molecule to exchange electrons with the environment. As expected from the molecular structure of these species, their electron donating ability is more important than their electron accepting character, again the Discodermin E peptide possessing an electron donating power larger than the others. On the basis of the previous definition and the scale established by these authors [55], it can be concluded that the Discodermins A–H peptides can be regarded as strong nucleophiles because the values for the Nucleophilicity N are greater than 3 eV in all the cases. As a summary of these results the following scales can be given for each of the considered descriptors: for the Electronegativity (χ), Discodermin E > Discodermin A > Discodermin D > Discodermin C > Discodermin B > Discodermin F > Discodermin H > Discodermin G; for the Hardness (η), Discodermin H > Discodermin G > Discodermin F > Discodermin B > Discodermin D > Discodermin C > Discodermin A > Discodermin E; for the Electrophilicity (ω), Discodermin E > Discodermin A > Discodermin D > Discodermin C > Discodermin B > Discodermin H > Discodermin F > Discodermin G; for the softness S it will be Discodermin E > Discodermin A > Discodermin B ≈ Discodermin C ≈ Discodermin E > Discodermin F > Discodermin G ≈ Discodermin H, for the Nucleophilicity N, Discodermin G > Discodermin C ≈ Discodermin F > Discodermin A ≈ Discodermin B > Discodermin D > Discodermin H > Discodermin E, while for the Electrodonating Power ($\omega^{-}$), Electroaccepting Power ($\omega^{+}$) and Net Electrophilicity ($\Delta \omega^{\pm }$) will be the same scale, namely Discodermin E > Discodermin A > Discodermin D > Discodermin B > Discodermin C > Discodermin F > Discodermin H > Discodermin G.

The presented global descriptors are a representation of the chemical reactivity of a molecule as a whole. However, local reactivity descriptors have been developed that can give an idea of the differences between the reactivity of each of the atoms that form the molecule. One of the most important groups of such reactivity descriptors are the Fukui functions [5,6,7] and the Dual Descriptor [59,60,61,62,63,64], which has been defined as:
Nucleophilic Fukui Function$f^{+}\left(r\right)=\rho_{N+1}\left(r\right)-\rho_{N}\left(r\right)$Electrophilic Fukui Function$f^{-}\left(r\right)=\rho_{N}\left(r\right)-\rho_{N-1}\left(r\right)$Dual DescriptorΔf(r) = ${\left(\frac{\partial \;f(r)}{\partial \;N}\right)}_{\upsilon (r)}$
which are relationships between the electronic densities of the neutral, positive and negative species as well as between the Nucleophilic and Electrophilic Fukui functions.

**Figure 5:**
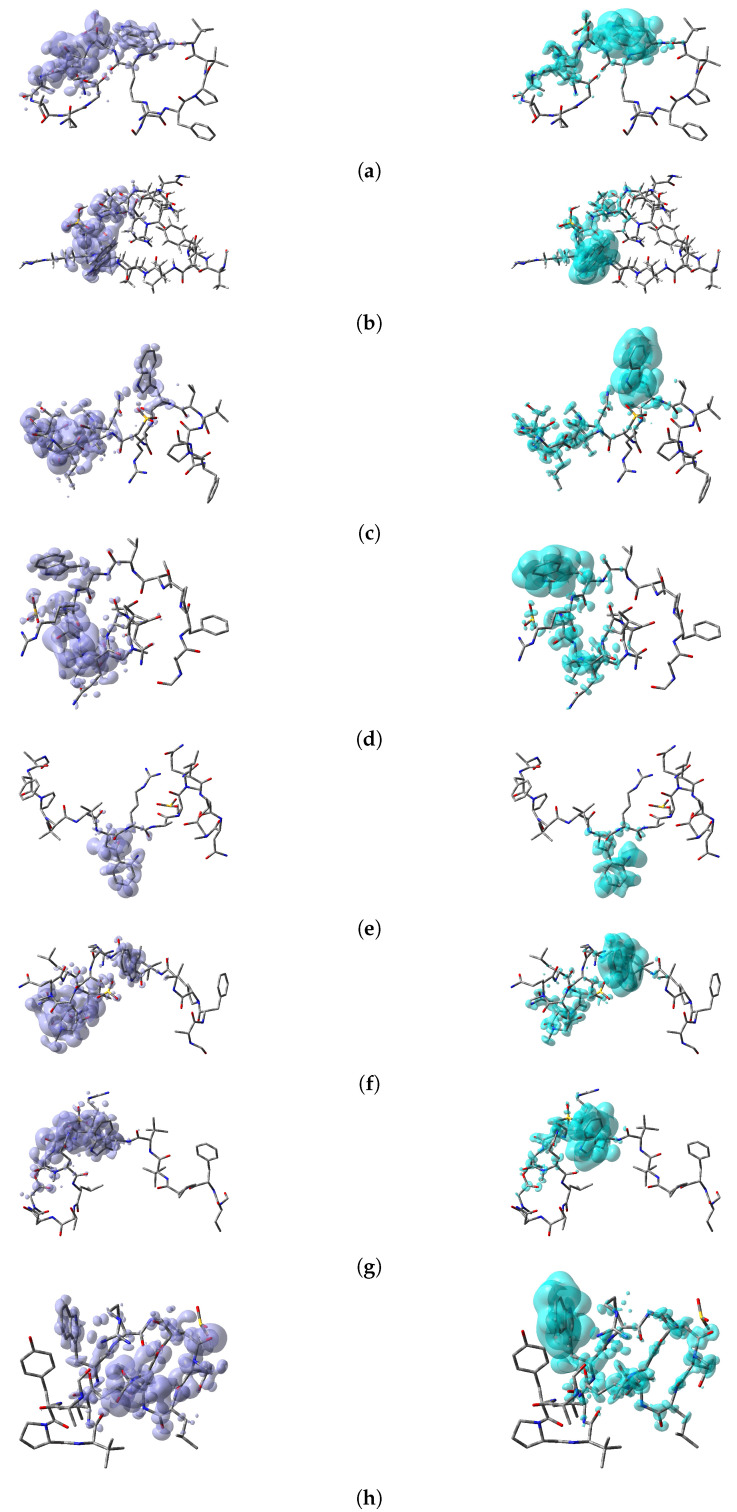
Graphical representation of the Dual Descriptor DD of the (**a**) Discodermin A, (**b**) Discodermin B, (**c**) Discodermin C, (**d**) Discodermin D, (**e**) Discodermin E, (**f**) Discodermin F, (**g**) Discodermin G and (**h**) Discodermin H molecules. Left: DD > 0, Right: DD < 0.

The Nucleophilic Fukui function, $f^{+}\left(r\right)$, reveals the sites on a molecular system that are susceptible to nucleophilic attacks and the Electrophilic Fukui function, $f^{+}\left(r\right)$, describes those sites that are more susceptible to electrophilic attacks. These local reactivity descriptors are very useful and have been used successfully for the identification of reactive sites. However, sometimes there is an overlap between the results of both descriptors and no conclusions can be accurately obtained. Instead, the Dual Descriptor Δf(r) or DD, can describe unambiguously nucleophilic and electrophilic sites within a molecule [64]. Thus, a graphical representation of the DD for the Discodermins A–H marine peptides is presented in Figure 5 showing clearly the areas within the molecules where DD > 0 and DD < 0 for a better understanding of the local chemical reactive of these molecules:

Another local reactivity descriptors are the Parr functions [65,66] that can be considered to be an alternative to the Fukui functions for describing sites or areas within the molecules where nucleophilic or electrophilic attacks will be favored. The Parr functions can be expressed as [65,66]:
Nucleophilic Parr Function$P^{-}\left(r\right)=\rho_{s}^{rc}\left(r\right)$Electrophilic Parr Function$P^{+}\left(r\right)=\rho_{s}^{ra}\left(r\right)$where $\rho_{s}^{rc}\left(r\right)$ and $\rho_{s}^{ra}\left(r\right)$ are related to the atomic spin density of the radical cation or anion of the considered system, respectively [58].

To perform a comparison between the results that can be obtained from both formulations, the predictions for the specific reactions sites coming from the Electrophilic and Nucleophilic Fukui functions analysis for each peptide have been compiled in a series of tables that are presented as Supplementary Materials in the form of an Appendix to this work together with the values that are the outcome of the Parr functions analysis. The Radical Fukui function $f^{0}\left(r\right)$, which can be considered to be an average of $f^{+}\left(r\right)$ and $f^{-}\left(r\right)$, and denotes the favorable sites for a radical attack, has also been included for the sake of completeness. In a complementary way to the tables, a graphical representation of these descriptors have been included as a series of figures such as the comparison between the results coming for both kinds of studies can be done accurately.

**Figure A1:**
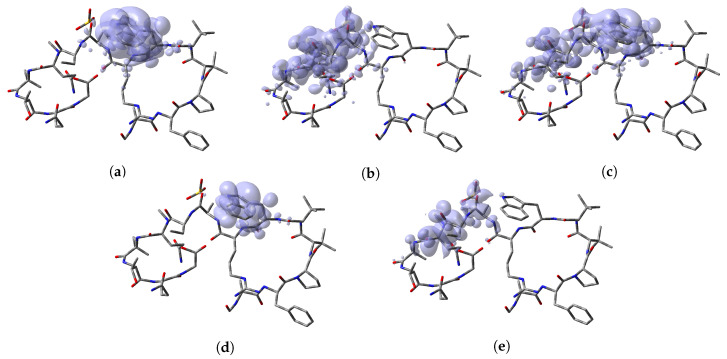
Graphical sketches of the local reactivity descriptors for Discodermin A showing the reactivity regions: (**a**) Electrophilic Fukui Function $f^{-}$, (**b**) Nucleophilic Fukui Function $f^{+}$, (**c**) Radical Fukui Function $f^{0}$, (**d**) Electrophilic Parr Function P−; and (**e**) Nucleophilic Parr Function P+.

**Figure A2:**
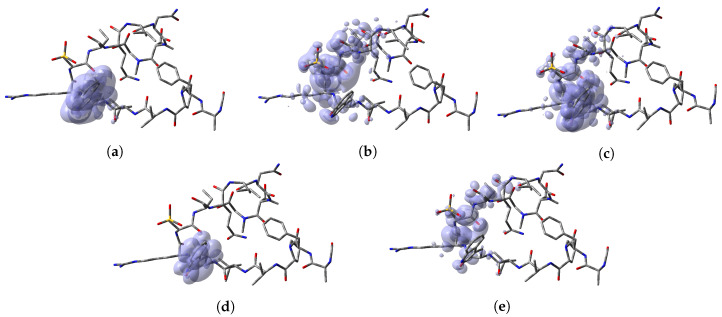
Graphical sketches of the local reactivity descriptors for Discodermin B showing the reactivity regions: (**a**) Electrophilic Fukui Function $f^{-}$, (**b**) Nucleophilic Fukui Function $f^{+}$, (**c**) Radical Fukui Function $f^{0}$, (**d**) Electrophilic Parr Function P−; and (**e**) Nucleophilic Parr Function P+.

**Figure A3:**
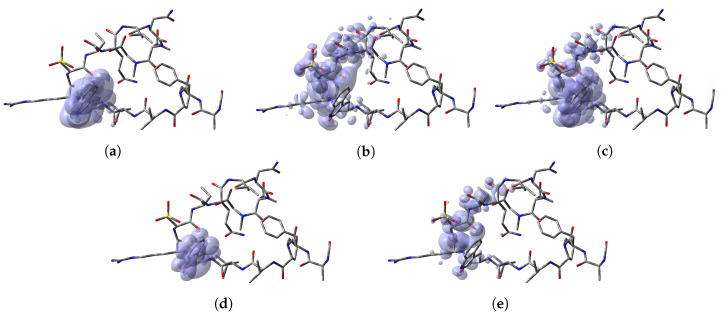
Graphical sketches of the local reactivity descriptors for Discodermin C showing the reactivity regions: (**a**) Electrophilic Fukui Function $f^{-}$, (**b**) Nucleophilic Fukui Function $f^{+}$, (**c**) Radical Fukui Function $f^{0}$, (**d**) Electrophilic Parr Function P−; and (**e**) Nucleophilic Parr Function P+.

**Figure A4:**
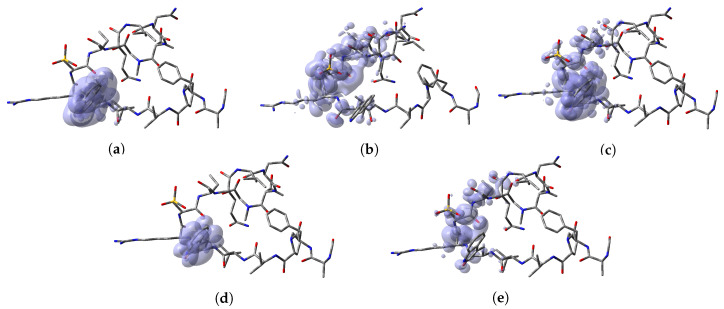
Graphical sketches of the local reactivity descriptors for Discodermin D showing the reactivity regions: (**a**) Electrophilic Fukui Function $f^{-}$, (**b**) Nucleophilic Fukui Function $f^{+}$, (**c**) Radical Fukui Function $f^{0}$, (**d**) Electrophilic Parr Function P−; and (**e**) Nucleophilic Parr Function P+.

**Figure A5:**
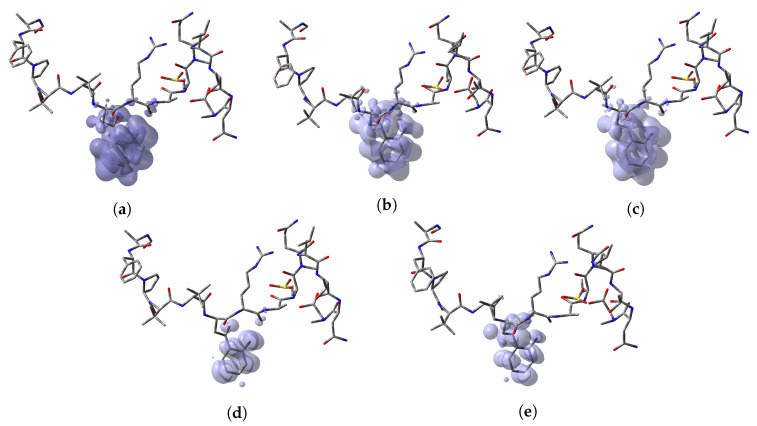
Graphical sketches of the local reactivity descriptors for Discodermin E showing the reactivity regions: (**a**) Electrophilic Fukui Function $f^{-}$, (**b**) Nucleophilic Fukui Function $f^{+}$, (**c**) Radical Fukui Function $f^{0}$, (**d**) Electrophilic Parr Function P−; and (**e**) Nucleophilic Parr Function P+.

**Figure A6:**
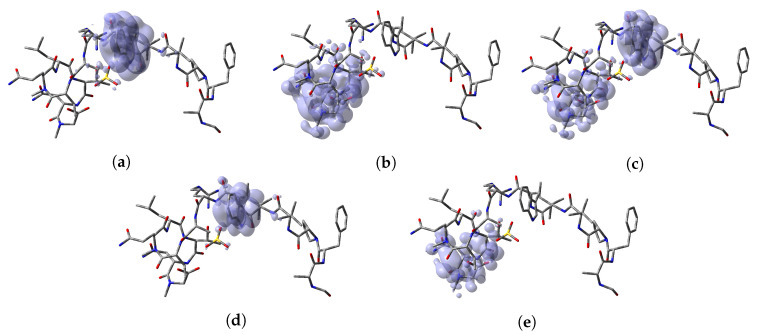
Graphical sketches of the local reactivity descriptors for Discodermin F showing the reactivity regions: (**a**) Electrophilic Fukui Function $f^{-}$, (**b**) Nucleophilic Fukui Function $f^{+}$, (**c**) Radical Fukui Function $f^{0}$, (**d**) Electrophilic Parr Function P−; and (**e**) Nucleophilic Parr Function P+.

**Figure A7:**
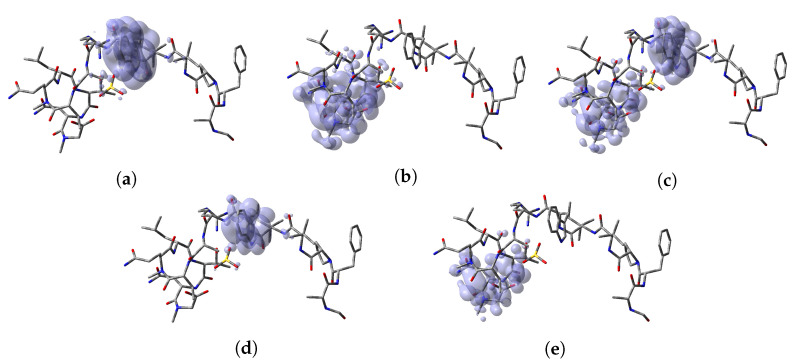
Graphical sketches of the local reactivity descriptors for Discodermin G showing the reactivity regions: (**a**) Electrophilic Fukui Function $f^{-}$, (**b**) Nucleophilic Fukui Function $f^{+}$, (**c**) Radical Fukui Function $f^{0}$, (**d**) Electrophilic Parr Function P−; and (**e**) Nucleophilic Parr Function P+.

**Figure A8:**
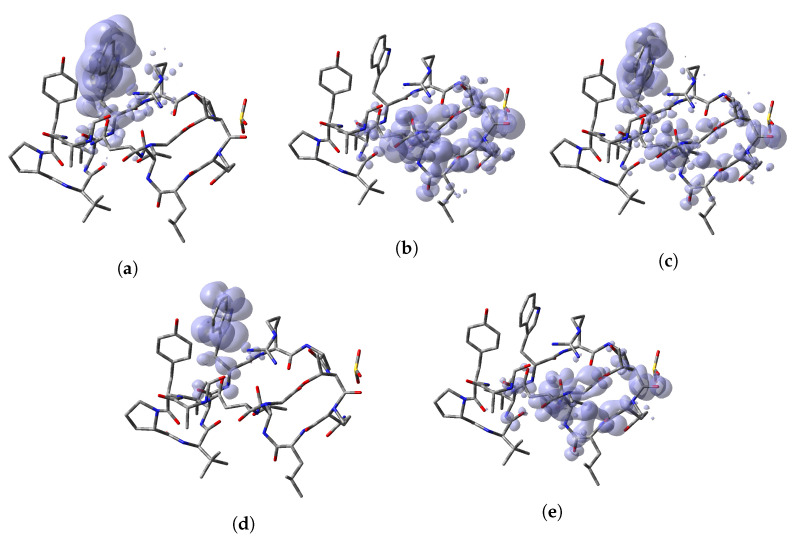
Graphical sketches of the local reactivity descriptors for Discodermin H showing the reactivity regions: (**a**) Electrophilic Fukui Function $f^{-}$, (**b**) Nucleophilic Fukui Function $f^{+}$, (**c**) Radical Fukui Function $f^{0}$, (**d**) Electrophilic Parr Function P−; and (**e**) Nucleophilic Parr Function P+.

By looking at the numerical and graphical results presented in Table A1, Table A2, Table A3, Table A4, Table A5, Table A6, Table A7, Table A8 and Figure A1, Figure A2, Figure A3, Figure A4, Figure A5, Figure A6, Figure A7, Figure A8 in Appendix A, it can be concluded that there is a nice agreement between the values coming from both formulations. In checking the numerical part, it can be seen that in all cases the reactive sites predicted by the Fukui functions or the Parr functions are the same. Indeed, the numerical values for the Parr functions are greater than for the Fukui functions with the meaning that the first ones are well defined. This observation is reinforced by checking at the graphical representations of the Fukui functions and the Parr functions, where although are extended over the same group of atoms or regions within the molecules, it can be seen that the Parr functions are more compact than the Fukui functions. Notwithstanding, this nice agreement could lead to the conclusion that if the Dual Descriptor DD can be built as the difference between both Fukui functions, a similar new reactivity descriptor could be defined in terms of the difference between both Parr functions giving the same information and being the starting point for future research on the field of chemical reactivity.

## 4. Conclusions

The Discodermins A–H family of cyclodepsipeptides of marine origin has been studied by resorting to some techniques of common use in the process of drug discovery and development showing that these kind molecules can be regarded as potential therapeutic drugs.

With this knowledge in mind, the chemical reactivity of the studied peptides has been exhaustively analyzed through the optimization of their structures using an MN12SX/Def2SVP/H2O model chemistry and the determination of their electronic properties by means of an improved model chemistry, namely MN12SX/Def2TZVP/H20, already used in previous works for the study of peptides, demonstrating their usefulness for this kind of calculations.

The chemical reactivity of the considered molecular systems was subject to an analysis based on a particular methodology developed by our research group named Conceptual DFT-based Computational Peptidology being the use of the MN12SX density functional validated once again by resorting to the KID procedure and the in-house software tool CDFT.

The analysis of the global and local reactivity descriptors arising from Conceptual DFT together with some proposals like the Nucleophilicity N and the Parr functions allowed for a complete understanding of the chemical reactivity of the studied peptides, by distinguishing the different chemical reactivities through the analysis of the global descriptors and the further identification of the reaction sites or regions within the molecules by resorting to Fukui and Parr functions as well as the Dual Descriptor.

Finally, a nice agreement was found between the outcome of the numerical and graphical analysis of the Fukui and Parr functions on each peptide leading to the conclusion that it could be possible to define a new chemical reactivity descriptor analog to the Dual Descriptor on the basis of the difference between the Parr functions which could act as an additional tool for the study of the chemical reactivity of molecular systems.

## Figures and Tables

**Table 1:** Predicted parameters useful for QSAR studies for the Discodermins A–H family of marine cyclodepsipeptides: ΔG of Solvation (in Kcal/mol), logP, TPSA (Å${}^{2}$) and Molecular Volume (Å${}^{3}$).

	ΔG of Solvation	logP	TPSA	Molecular Volume
Discodermin A	−96.29	−5.51	657.09	1537.74
Discodermin B	−105.04	−5.37	648.30	1521.64
Discodermin C	−93.16	−5.37	648.30	1521.64
Discodermin D	−88.03	−5.50	648.30	1505.40
Discodermin E	−103.39	−5.71	677.66	1539.28
Discodermin F	−112.03	−5.41	650.36	1554.79
Discodermin G	−108.39	−5.41	650.36	1554.79
Discodermin H	−92.26	−5.59	670.59	1546.00

**Table 2:** Absorption, Distribution, Metabolism, and Excretion (ADME) parameters related to Discodermin A–H pharmacokinetics.

	**Discodermin A**	**Discodermin B**	**Discodermin C**	**Discodermin D**
GI absorption	Low	Low	Low	Low
BBB permeant	No	No	No	No
P-gp substrate	Yes	Yes	Yes	Yes
CYP1A2 inhibitor	No	No	No	No
CYP2C19 inhibitor	No	No	No	No
CYP2C9 inhibitor	No	No	No	No
CYP2D6 inhibitor	No	No	No	No
CYP3A4 inhibitor	No	No	No	No
Log K${}_{p}$	−17.57 cm/s	−17.88 cm/s	−17.88 cm/s	−18.08 cm/s
(skin permeation)				
	**Discodermin E**	**Discodermin F**	**Discodermin G**	**Discodermin H**
GI absorption	Low	Low	Low	Low
BBB permeant	No	No	No	No
P-gp substrate	Yes	Yes	Yes	Yes
CYP1A2 inhibitor	No	No	No	No
CYP2C19 inhibitor	No	No	No	No
CYP2C9 inhibitor	No	No	No	No
CYP2D6 inhibitor	No	No	No	No
CYP3A4 inhibitor	No	No	No	No
Log K${}_{p}$	−18.09 cm/s	−17.26 cm/s	−17.26 cm/s	−17.78 cm/s
(skin permeation)				

**Table 3:** HOMO, LUMO and SOMO orbital energies, HOMO-LUMO gap and the KID descriptors (all in eV) tested in the verification of the Koopmans-like behavior of the MN12SX density functional for the Discodermins A–H family of marine cyclodepsipeptides.

	HOMO	LUMO	SOMO	H-L Gap	*J*(*I*)	*J*(*A*)	*J*(*HL*)	ΔSL
Discodermin A	−5.6567	−1.5755	−1.5356	4.0812	0.048	0.013	0.050	0.040
Discodermin B	−5.6589	−1.4055	−1.3577	4.2534	0.039	0.022	0.045	0.048
Discodermin C	−5.6347	−1.3674	−1.5245	4.2673	0.048	0.013	0.050	0.040
Discodermin D	−5.6793	−1.4667	−1.4257	4.2126	0.044	0.018	0.047	0.041
Discodermin E	−5.8352	−2.1002	−2.1114	3.7350	0.002	0.007	0.007	0.011
Discodermin F	−5.6423	−1.3260	−1.3223	4.3163	0.060	0.005	0.060	0.003
Discodermin G	−5.5677	−0.9766	−0.9822	4.5911	0.047	0.003	0.047	0.005
Discodermin H	−5.7182	−1.1391	−1.1123	4.5791	0.051	0.019	0.054	0.029

**Table 4:** Global reactivity descriptors for the Discodermin family of marine cyclopeptides: Electronegativity (χ), Hardness (η), Electrophilicity (ω) (all in eV), Softness S (in eV${}^{-1}$), Nucleophilicity N, Electrodonating Power ($\omega^{-}$), Electroaccepting Power ($\omega^{+}$) and Net Electrophilicity ($\Delta \omega^{\pm }$) (also in eV).

	χ	η	γ	S	N	$\omega^{-}$	$\omega^{+}$	$\Delta \omega^{\pm }$
Discodermin A	3.6339	4.1418	1.5941	0.2414	3.4645	5.2672	1.6511	6.9183
Discodermin B	3.5410	4.3143	1.4531	0.2318	3.4622	4.9652	1.4330	6.3981
Discodermin C	3.5524	4.2590	1.4816	0.2348	3.4865	4.8896	1.3885	6.2781
Discodermin D	3.5858	4.2747	1.5040	0.2339	3.4420	5.0803	1.5073	6.5876
Discodermin E	3.9703	3.7254	2.1157	0.2584	3.2860	6.4321	2.4644	8.8966
Discodermin F	3.5118	4.3812	1.4075	0.2283	3.4789	4.8243	1.3401	6.1644
Discodermin G	3.2971	4.6348	1.1728	0.2158	3.5535	4.2552	0.9830	5.2382
Discodermin H	3.4446	4.6494	1.2760	0.2151	3.4029	4.5677	1.1391	5.7068

## Abbreviations

The following abbreviations are used in this manuscript:

DFT	Density Functional Theory
HOMO	Highest Occupied Molecular Orbital
LUMO	Lowest Unoccupied Molecular Orbital
SOMO	Single Occupied Molecular Orbital
KID	Koopmans in DFT
SMD	Solvation Model Density
QSAR	Quantitative Structure Activity Relationships
IEF-PCM	Integral Equation Formalism-Polarized Continuum Model
IP	Ionization Potential Energy
EA	Electronic Affinity
DFTBA	Density Functional Tight-Binding Approximation
DD	Dual Descriptor

## Appendix A

**Table A1:** Condensed local reactivity descriptors for Discodermin A denoting the reactivity sites: Electrophilic Fukui Function $f^{-}$, Nucleophilic Fukui Function $f^{+}$, Radical Fukui Function $f^{0}$, Electrophilic Parr Function P− and Nucleophilic Parr Function P+.

	$f^{-}$	$f^{+}$	$f^{0}$	P−	P+
Discodermin A	C72 (0.1172)	O12 (0.1326)	C72 (0.0574)	C72 (0.2415)	O12 (0.1615)

**Table A2:** Condensed local reactivity descriptors for Discodermin B denoting the reactivity sites: Electrophilic Fukui Function $f^{-}$, Nucleophilic Fukui Function $f^{+}$, Radical Fukui Function $f^{0}$, Electrophilic Parr Function P− and Nucleophilic Parr Function P+.

	$f^{-}$	$f^{+}$	$f^{0}$	P−	P+
Discodermin B	C71 (0.1243)	O8 (0.0961)	C71 (0.0622)	C71 (0.2455)	O8 (0.1091)

**Table A3:** Condensed local reactivity descriptors for Discodermin C denoting the reactivity sites: Electrophilic Fukui Function $f^{-}$, Nucleophilic Fukui Function $f^{+}$, Radical Fukui Function $f^{0}$, Electrophilic Parr Function P− and Nucleophilic Parr Function P+.

	$f^{-}$	$f^{+}$	$f^{0}$	P−	P+
Discodermin C	C75 (0.1187)	O14 (0.0694)	C71 (0.0594)	C75 (0.2388)	O14 (0.0779)

**Table A4:** Condensed local reactivity descriptors for Discodermin D denoting the reactivity sites: Electrophilic Fukui Function $f^{-}$, Nucleophilic Fukui Function $f^{+}$, Radical Fukui Function $f^{0}$, Electrophilic Parr Function P− and Nucleophilic Parr Function P+.

	$f^{-}$	$f^{+}$	$f^{0}$	P−	P+
Discodermin D	C74 (0.1136)	O11 (0.1024)	C74 (0.0572)	C74 (0.2401)	O11 (0.1450)

**Table A5:** Condensed local reactivity descriptors for Discodermin E denoting the reactivity sites: Electrophilic Fukui Function $f^{-}$, Nucleophilic Fukui Function $f^{+}$, Radical Fukui Function $f^{0}$, Electrophilic Parr Function P− and Nucleophilic Parr Function P+.

	$f^{-}$	$f^{+}$	$f^{0}$	P−	P+
Discodermin E	N98 (0.1774)	C90 (0.1439)	N98 (0.1069)	N98 (0.3879)	C90 (0.2596)

**Table A6:** Condensed local reactivity descriptors for Discodermin F denoting the reactivity sites: Electrophilic Fukui function $f^{-}$, Nucleophilic Fukui function $f^{+}$, Radical Fukui Function $f^{0}$, Electrophilic Parr Function P− and Nucleophilic Parr Function P+.

	$f^{-}$	$f^{+}$	$f^{0}$	P−	P+
Discodermin F	C73 (0.1123)	O41 (0.1002)	C73 (0.0562)	C73 (0.2339)	O41 (0.1346)

**Table A7:** Condensed local reactivity descriptors for Discodermin G denoting the reactivity sites: Electrophilic Fukui Function $f^{-}$, Nucleophilic Fukui function $f^{+}$, Radical Fukui Function $f^{0}$, Electrophilic Parr Function P− and Nucleophilic Parr Function P+.

	$f^{-}$	$f^{+}$	$f^{0}$	P−	P+
Discodermin G	C116 (0.1128)	C14 (0.1227)	C116 (0.0580)	C116 (0.8123)	C14 (0.2565)

**Table A8:** Condensed local reactivity descriptors for Discodermin H denoting the reactivity sites:Electrophilic Fukui Function $f^{-}$, Nucleophilic Fukui function $f^{+}$, Radical Fukui Function $f^{0}$, Electrophilic Parr Function P− and Nucleophilic Parr Function P+.

	$f^{-}$	$f^{+}$	$f^{0}$	P−	P+
Discodermin H	C119 (0.1132)	C20 (0.0.0629)	C116 (0.0396)	C119 (0.2316)	C20 (0.1191)

## Notes

**Sample Availability:** Samples of the compounds are not available from the authors.
